# Histopathology of brain AVMs part I: microhemorrhages and changes in the nidal vessels

**DOI:** 10.1007/s00701-020-04391-w

**Published:** 2020-05-12

**Authors:** Patrik Järvelin, Roosa Wright, Henri Pekonen, Sara Keränen, Tuomas Rauramaa, Juhana Frösen

**Affiliations:** 1 Hemorrhagic Brain Pathology Research Group, NeuroCenter, Kuopio University Hospital, Kuopio, Finland; 2 Dept of Molecular Medicine, AIV-Institute, University of Eastern Finland, Kuopio, Finland; 3 Dept of Pathology, Kuopio University Hospital, Kuopio, Finland; 4 Hemorrhagic Brain Pathology Research Group, University of Tampere, Tampere, Finland; 5 Dept of Neurosurgery, Tampere University Hospital, Teiskontie 35, PO Box 33521, Tampere, Finland

**Keywords:** Arteriovenous malformation, Rupture, Microhemorrhage, Vascular degeneration, Inflammation, Brain

## Abstract

**Background:**

Arteriovenous malformations of the brain (bAVM) may rupture from aneurysms or ectasias of the feeding, draining, or nidal vessels. Moreover, they may rupture from the immature, fragile nidal vessels that are characteristic to bAVMs. How the histopathological changes of the nidal vessels associate with clinical presentation and hemorrhage of the lesion is not well known.

**Materials and methods:**

We investigated tissue samples from surgically treated bAVMs (*n* = 85) using standard histological and immunohistochemical stainings. Histological features were compared with the clinical presentation of the patient.

**Results:**

Microhemorrhages from nidal vessels were found both in bAVMs with a history of clinically evident rupture and in bAVMs considered unruptured. These microhemorrhages were associated with presence of immature, pathological nidal vessels (*p* = 0.010) and perivascular inflammation of these vessels (*p* = 0.001), especially with adhesion of neutrophils (*p* < 0.001). In multivariate analysis, perivascular inflammation (OR = 19, 95% CI 1.6 to 230), neutrophil infiltration of the vessel wall (OR = 13, 95% CI 1.9 to 94), and rupture status (OR = 0.13, 95% CI 0.017 to 0.92) were significantly associated with microhemorrhages.

**Conclusions:**

Clinically silent microhemorrhages from nidal vessels seem to be very common in bAVMs, and associate with perivascular inflammation and neutrophil infiltration. Further studies on the role of perivascular inflammation in the clinical course of bAVMs are indicated.

**Electronic supplementary material:**

The online version of this article (10.1007/s00701-020-04391-w) contains supplementary material, which is available to authorized users.

## Introduction

Arteriovenous malformations of the brain (bAVMs) are high-flow vascular malformations which allow direct connections between cerebral arteries and veins [11]. BAVMs are a major cause of intracranial hemorrhage in the younger population [10], but unruptured bAVMs may also cause other symptoms, such as epileptic seizures and headache [12, 13]. BAVMs are relatively rare with an incidence of approximately 1 per 100,000 person years [1], and they often remain undiagnosed until rupture and subsequent intracerebral hemorrhage (ICH). Although multiple genetic polymorphisms can predispose to the dysregulation of angiogenesis leading to the development of bAVMs, recent reports suggest that in most sporadic bAVMs, the presence of activating somatic KRAS mutations explains the development of the lesion [4, 8, 9].

**Fig. 1:**
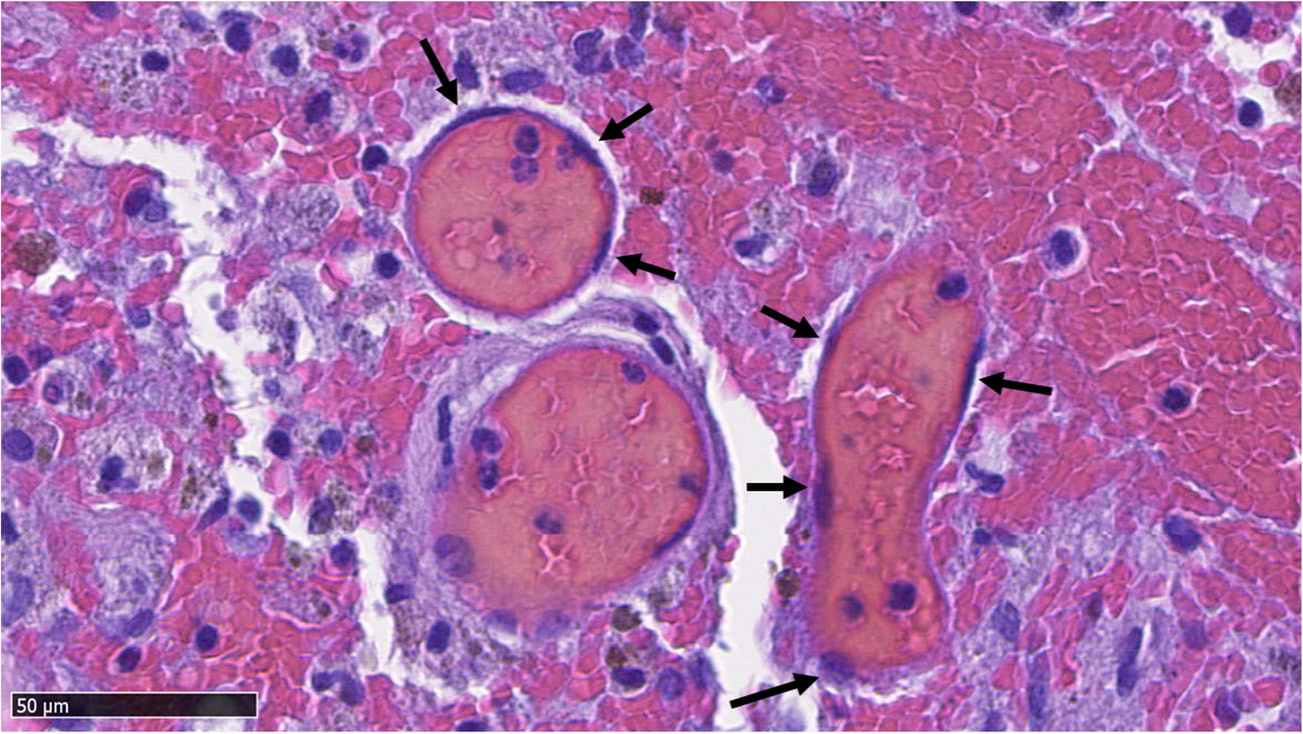
An abnormal vessel typical to bAVMs. The structure of the vessel resembles a capillary with no smooth muscle in spite of diameter much larger than that of a capillary. The endothelial layer is marked with arrows and the lumen with *

**Fig. 2:**
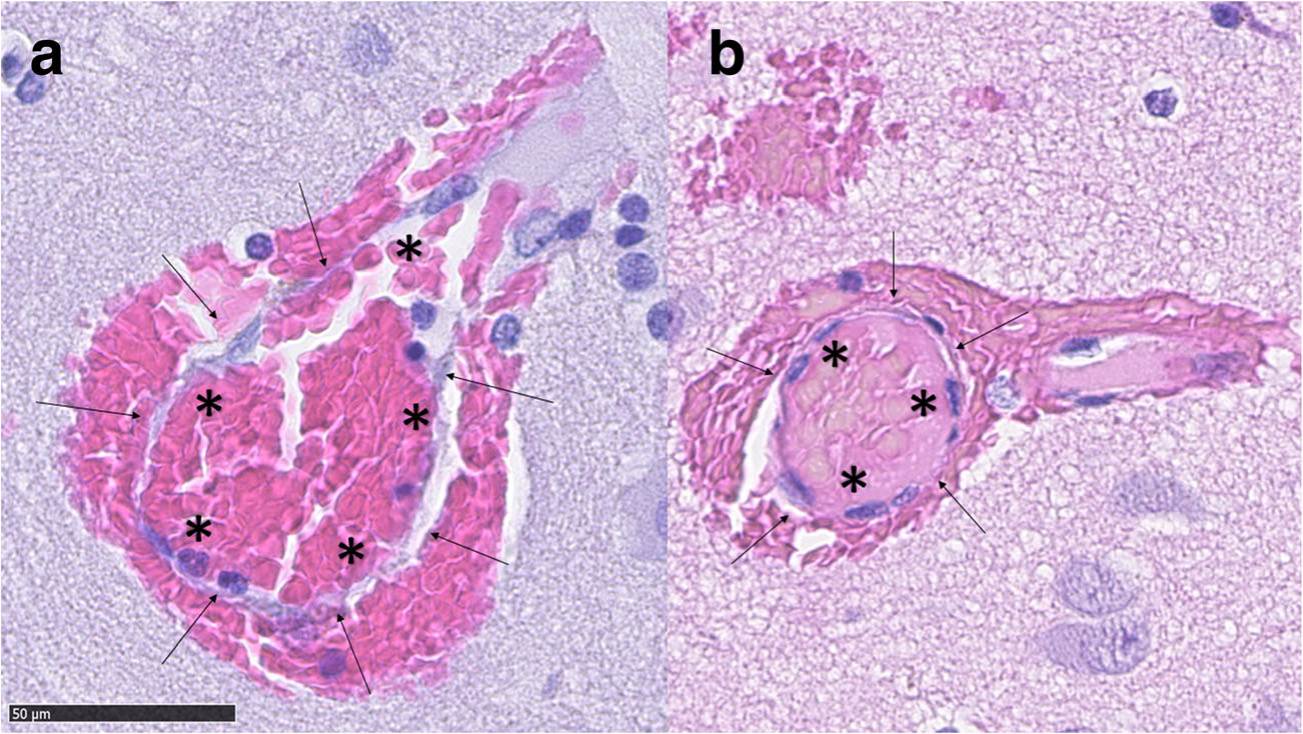
Two small bAVM vessels which have bled into their immediate surroundings. The endothelium of the vessels (marked with arrows) is thin in comparison with the size of the lumen (marked with asterisks). A typical example of microhemorrhage in bAVM samples

**Fig. 3:**
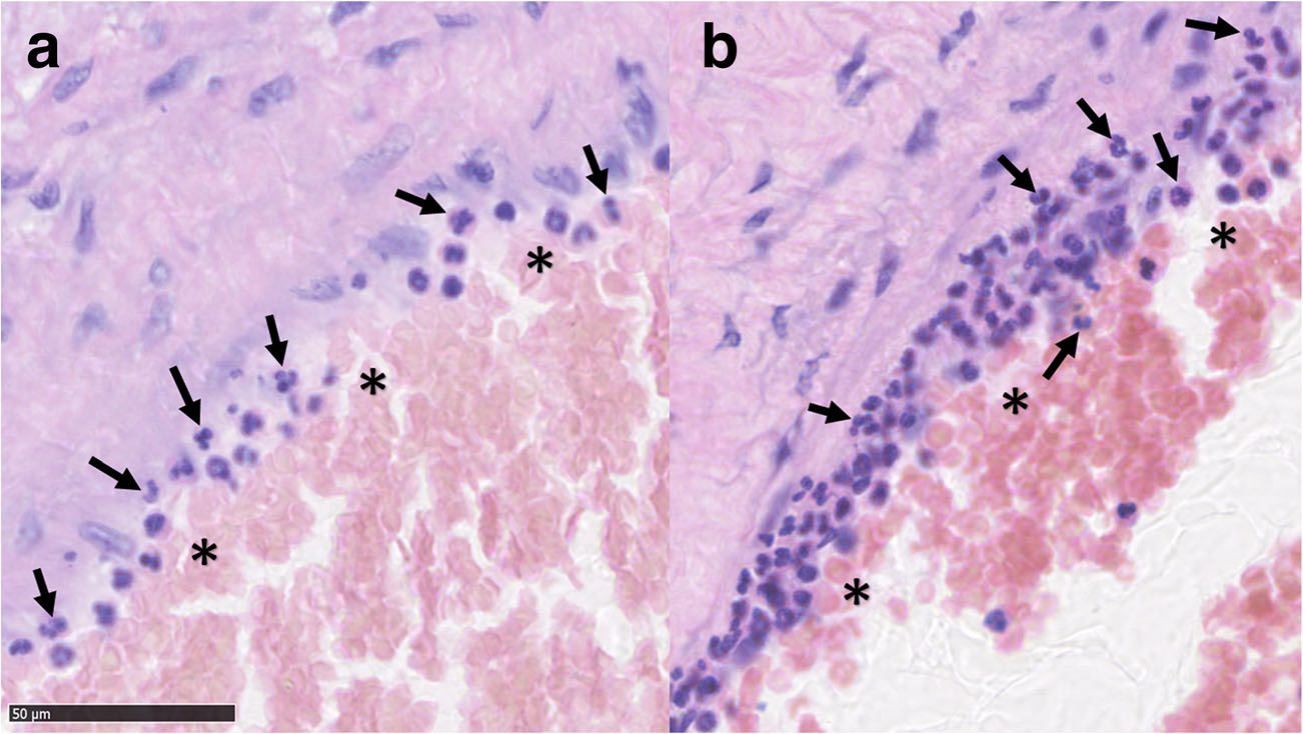
Light (**a**) and strong (**b**) recruitment and adhesion of neutrophils (marked with arrows) to the luminal surface (marked with asterisks) of a bAVM vessel and the vessel wall

**Fig. 4:**
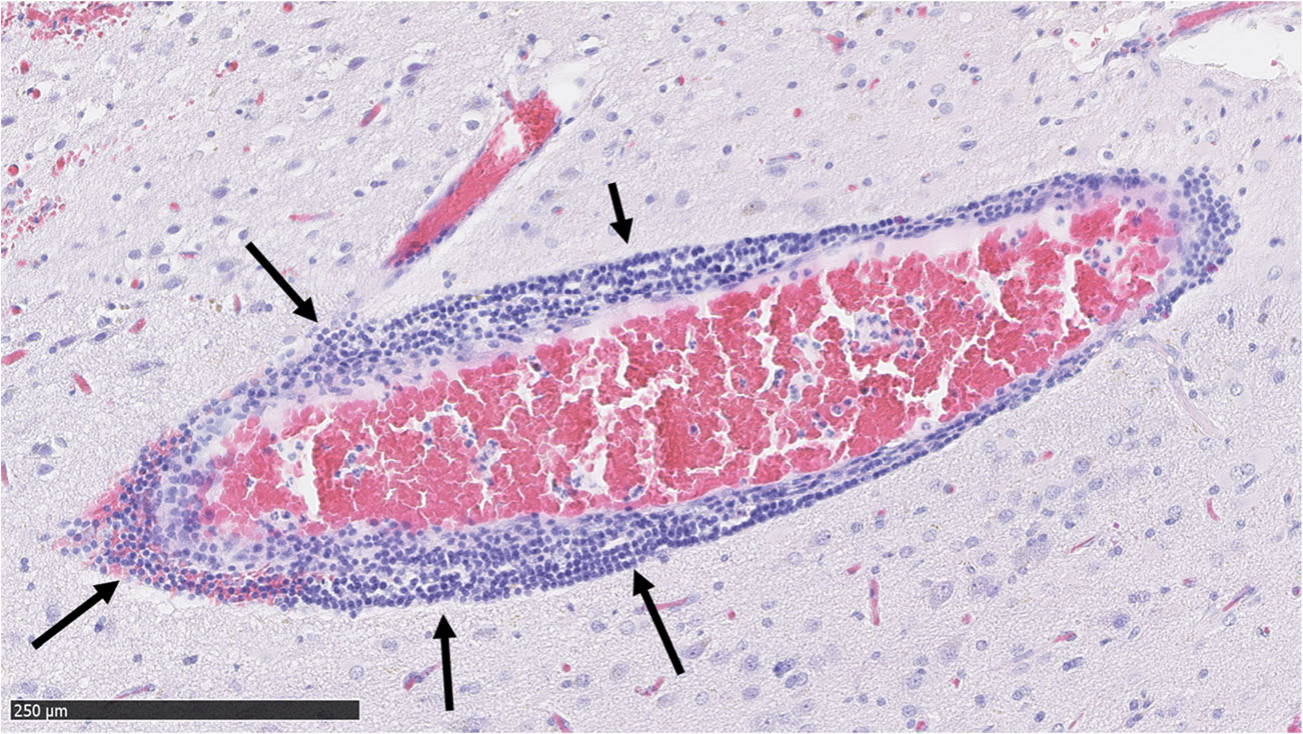
Dense perivascular inflammation (marked with arrows) around a bAVM vessel. The inflammation consists of mononuclear lymphocytes

**Fig. 5:**
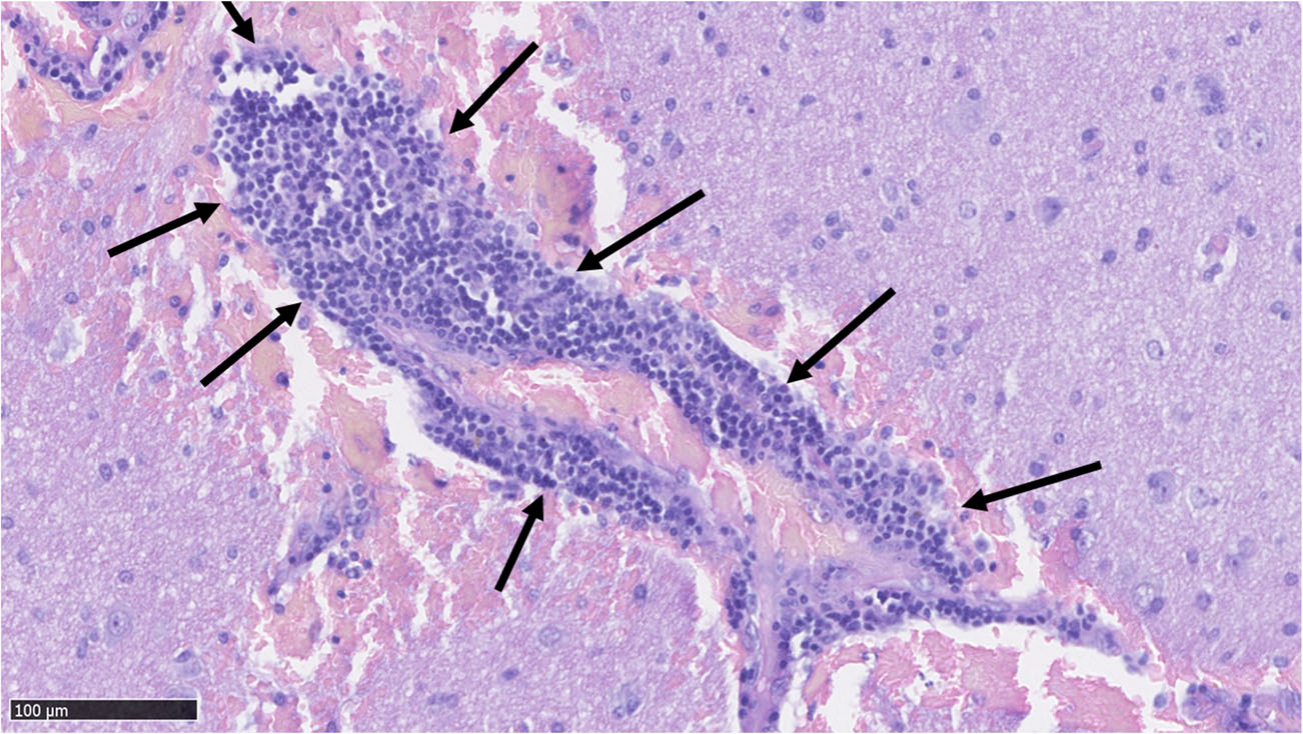
A bAVM vessel with a high degree of perivascular mononuclear inflammation (marked with arrows). The vessel has also hemorrhaged into its immediate surroundings

Macroscopically, bAVMs are tangles of abnormally enlarged vessels which directly shunt blood from the arterial system to the venous system due to a lack of an intervening capillary bed [4]. Histologically, these abnormal vessels resemble capillaries despite their diameter, which is multiple times larger than that of a capillary (Figs. 1, 2, 3, 4, and 5). The lack of a normal capillary bed causes abnormally high flow through these nidal vessels due to the subsequent lack of normal capillary bed resistance [4]. Since blood vessels adapt to changes in flow through flow-induced vessel wall remodeling [3], this abnormally high flow often causes ectatic (expansive, enlarging) remodeling of the veins draining the bAVM or of the arteries feeding it which, in addition to causing enlargement of the vessel caliber, may lead to formation of venous ectasias, intranidal aneurysms, or aneurysms of the feeding arteries.

Venous ectasia, intranidal aneurysms, and aneurysms of the arteries feeding the bAVM are thought to represent weakened parts of the bAVM vasculature, encompassing an increased risk of rupture [5]. However, according to common surgical experience, the pathological nidal vessels are also abnormally fragile. Moreover, already in 1979, Drake reported that many AVMs considered asymptomatic demonstrated in fact at surgery signs of prior hemorrhage around the nidus, suggesting that fragile nidal vessels are an underestimated source hemorrhage [2]. While the macroscopic remodeling of the bAVM vasculature which manifests as a venous ectasias, intranidal aneurysms, and aneurysms of the feeding arteries can be diagnosed with the computer tomography angiography (CTA) or digital substraction angiography (DSA) with which the bAVM was diagnosed, neither modality gives information on the condition and risk of rupture of the nidal vessels. Magnetic resonance imaging (MRI), which is commonly used in the diagnostic workup of bAVMs, may indirectly point to fragility of the nidal vessels through showing signs of microhemorrhage (hemosiderin) in the nidal region [6].

Although nidal microhemorrhages clearly indicate fragility of the nidal vessels, whether such microhemorrhages indicate an increased risk of later fulminant rupture is not well established. Neither is it known to which kind of histological changes or clinical factors these microhemorrhages associate to. We investigated in tissue samples of surgically treated bAVMs the histological changes associated with signs of prior or recent microhemorrhages, as well as their clinical correlates.

## Materials and methods

### Tissue collection and patient data

A total of 85 formalin-fixed paraffin-embedded (FFPE) bAVM tissue samples were collected from surgically treated patients in Kuopio University Hospital (KUH). Clinical information from the patients including, i.a., age, sex, bAVM rupture status, and epileptic symptoms, was collected from patient records. In addition, several variables were assessed based on the histological sections. A summary of patient demographics and of the clinical presentation is given in Table 1.

**Table 1:** Association of patient demographics and clinical presentation with histologically observed microhemorrhages

Clinical variables	Microhemorrhages present		*P* value
	Yes (*n* = 72)	No (*n* = 13)	
Age	33y (4–67)	40.5 years (9–73)	0.629
Females	44% (32/72)	31% (4/13)	0.358
Prior fulminant rupture	56% (40/72)	77% (10/13)	0.150
Prior embolization	60% (43/72)	23% (3/13)	0.015
Prior radiotherapy	8.3% (6/72)	7.7% (1/13)	0.938

### Immunohistochemistry

The FFPE bAVM tissue samples were cut to 4-μm sections that were deparaffinized and rehydrated through the standard procedures. These sections were subjected to hematoxylin & eosin, hematoxylin, and glycophorin stainings. For the glycophorin immunostaining, after being deparaffinized, the sections underwent antigen-retrieval in heated citrate buffer (pH 6), followed by serum block in 3% normal horse serum in PBS. After serum block, the sections were incubated with a mouse monoclonal anti-glycophorin antibody (clone JC159, Novus Biologicals, Abingdon, UK) diluted 1:100 in 1.5% normal horse serum in PBS at 4 °C overnight. Following 3 × 5 min PBS washes, the sections were incubated 30 min at room temperature with a biotinylated anti-mouse secondary antibody (Vectastain, Vector, Burlingame, CA, USA; 1:200 dilution). After this, sections underwent 3 × 5 min washes in PBS, a 20-min endogenous peroxidase block with 3% H2O2 in PBS, a second 3 × 5 min wash in PBS, followed by 30-min incubation with horseradish peroxidase conjugated avidin-biotin complex. Peroxidase activity signifying bound primary antibody was detected with diaminobenzidine (DAB). The sections were counterstained with hematoxylin and mounted with Depex after dehydration. Sections with primary antibody omitted were used as negative controls.

### Histological analysis

Immunoperoxidase-stained tissue sections were scanned with a digital slide scanner (Nanozoomer XR, Hamamatsu, city, Japan) following which all the scanned sections underwent histological analysis using NDP.view2 software and up to × 80 magnification when necessary. The samples were studied by three independent observers (PJ, HP, RW). A consensus score was attained and reviewed by a neuropathologist (TR).

Definitions for the histological scores are presented in supplementary Tables 1 and 2.

### Statistics

For nominal and ordinal variables, percentages and proportions are given and chi-square test used for statistical comparison. For continuous variables, median and range are given and Mann-Whitney *U* test used for statistical comparison. Logistic regression with backward selection was used for multivariate analysis. Statistics were calculated with SPSS 22.0 software (IBM Corp., Armonk, NY, USA).

## Results

### Microhemorrhages do not associate with fulminant rupture

We found no association between microhemorrhages and prior fulminant rupture (*p* = 0.150, Table 1) or hemosiderin and prior fulminant rupture (*p* = 0.722). However, the strong association observed between blood in the parenchyma and microhemorrhage (*p* = 0.000) suggests that the small ruptures in the nidal vessels might be a bigger contributor to the overall presence of blood in bAVM samples than previously thought. Microhemorrhage and immature vessels were associated (*p* = 0.010, Tables 1 and 2), implying that the pathological bAVM vessels are prone to smaller ruptures and a possible source for the blood in the parenchyma.

**Table 2:** Association of histological variables with histologically observed microhemorrhages in the KUH cohort

Histological variable	Microhemorrhages present		*P* value
	Yes (*n* = 73)	No (*n* = 12)	
Immature vessels	84.7% (61/72)	53.8% (7/13)	0.010
Hyalinized vessels	18.1% (13/72)	23.1% (3/13)	0.670
Perivascular inflammation	56.9% (41/72)	7.69% (1/13)	0.001
Neutrophil infiltration of the vessel walls	68.1% (49/72)	15.4% (2/13)	0.000
Hemosiderin (median and range)	1 (0–3)	1 (0–3)	0.831
Inflammation (median and range)	2 (1–3)	2 (1–3)	0.726
Macrophages	78% (56/72)	77% (10/13)	0.946
Neutrophils	82% (59/72)	77% (10/13)	0.670

### Microhemorrhages associate with perivascular inflammation

Microvascular hemorrhage associated strongly with both mononuclear perivascular inflammation and neutrophil infiltration of the vessel walls (*p* values of 0.001 and 0.000, respectively). Mononuclear perivascular inflammation was found in 49.4% (42/85) of the samples (Table 2). In addition to mononuclear inflammatory cells, perivascular neutrophil infiltration was also seen, including adhesion of neutrophils to the luminal surfaces of bAVM nidal vessels. This phenomenon (neutrophil infiltration of the vessel walls) was observed in 60% (51/85) of the samples (Table 2). No association was found between microvascular hemorrhage and inflammation of the parenchyma (*p* = 0.726, Table 2) or neutrophil infiltration of the vessel walls and inflammation of the parenchyma (*p* = 0.547).

Interestingly, we found proportionally more (93.5% vs 74.4%) microhemorrhages in AVMs embolized before surgery than in non-embolized samples. Embolized AVMs were also more likely (63.0% vs 33.3%) to have perivascular inflammation.

### Association of microhemorrhages with clinical variables in multivariate analysis

We used two logistic regression models with backward selection to evaluate possible independent risk factors for microhemorrhage in bAVMs. The first model included embolization and inflammation in the parenchyma and rupture status as explaining variables, with embolization remaining significant in the model (OR = 4.9, 95% CI 1.3 to 20). The second model included age, gender, rupture status, embolization, perivascular inflammation, neutrophil infiltration, and immature vessels. In this model, perivascular inflammation (OR = 19, 95% CI 1.6 to 230), neutrophil infiltration of the vessel wall (OR = 13, 95% CI 1.9 to 94), and rupture status (OR = 0.13, 95% CI 0.017 to 0.92) were significant.

## Discussion

In our series, intranidal microhemorrhages were very common in both ruptured bAVMs and clinically unruptured bAVMs. Hemosiderin, a sign of prior hemorrhage, was also very frequently found. These results show that many bAVMs deemed stable and unruptured based on the clinical history of the patient have in fact experienced subclinical hemorrhages, which is likely to have an impact on the pathophysiology and untreated clinical course of the lesion. Our results confirm with histology the conclusion that Drake had made in 1979 based on observations made during surgery of 166 bAVMs [2].

We found no association between microhemorrhages and history of clinically evident rupture. However, our data is in line with the hypothesis that the pathological nidal vessels of bAVMs are prone to smaller ruptures (microhemorrhage). Considering this, it is not surprising though of great interest to note that embolization prior to surgery is associated with an increased presence of microhemorrhages. Due to partial obstruction of the bAVM nidus, embolization likely increases blood pressure elsewhere in the nidus, predisposing the remaining functional parts of the nidus to rupture and causing inflammation of the nidal vessel walls. What is somewhat suprising is that the presence of degenerated, hyalinized vessels described in bAVMs by McCormick already in 1966 [7] did not clearly associate with microhemorrhages. This might be due to the fact that also non-hyalinized nidal vessels had hemorrhage.

Considering this, another interesting observation is the high prevalence of perivascular inflammation (48.2%, 41/85 of the samples) and the strong association between perivascular inflammation and microhemorrhages. The high prevalence of nidal vessel inflammation observed in bAVM samples and its association with microhemorrhage (a sign of weakened vessel wall structure) suggest that the role of perivascular and perinidal inflammation in the pathobiology and evolution of bAVMs should be studied further. In particular, the observed association of neutrophil adhesion and infiltration of the bAVM vessels with microhemorrhages is of great interest considering the role and etiology of inflammation in the pathobiology of bAVMs.

### Limitations of the study

All of our samples were from surgically treated patients, which may introduce selection bias to our results. The possibility of our microhemorrhage scoring being flawed in samples with large amounts of blood due to a history of clinically evident macroscopic rupture is a valid concern, but the fact that we observed more microhemorrhages in samples without a history of clinical rupture (91.4%, 32/35) than in samples with a history of rupture (80%, 40/50) suggests that this is not the case. In multivariate logistic regression analysis with backward selection, blood in the parenchyma loses its significance as an explaining variable for microhemorrhage.

Treatment with surgical instruments might also have caused additional blood cells in the parenchyma to be mistaken for microhemorrhage. However, the strong association observed between microhemorrhages and immature vessels in the sample implies that microhemorrhages are indeed a result of the fragility of the immature bAVM vessels, rather than a result of surgical trauma.

### Conclusion

Microhemorrhages appear to be more common in bAVMs than previously thought. The prevalence of nidal vessel inflammation in bAVMs is high, and it seems to be a risk factor for microhemorrhage. Further studies are indicated to investigate the cause of perivascular inflammation in bAVMs as well as its role in the evolution and risk of rupture of bAVMs.

## Electronic supplementary material

ESM 1 (DOCX 14 kb).
